# The impact of cultural origin on the psychiatric expertise in Switzerland: a focus on sexual violence illustrated by two criminal cases

**DOI:** 10.3389/fpsyt.2024.1390224

**Published:** 2025-01-28

**Authors:** Marco De Pieri, Neva Suardi

**Affiliations:** ^1^ Department of Psychiatry, University Hospitals of Geneva, Geneva, Switzerland; ^2^ Cabinet Toepffer, Rue Rodolphe-Toepffer 8, Genève, Switzerland

**Keywords:** psychiatric expertise, sexual violence, ethnopsychiatry, acculturation, migration

## Abstract

Cultural biases and integration in novel socio-geographic contexts are relevant factors for the understanding of dynamics beneath sexual violence, and possibly play a role in modifying responsibility and perpetrators treatment. Here we offer a conceptual analysis of the relevant literature and two case study. Cultural factors influence power dynamics and individual values, impacting the occurrence of sexual violence; the understanding of “coercion” varies across cultures, and cultural legitimization may ensue. The consequences of sexual assault also differ, with shame prevailing in socio-centric societies and guilt in ego-centric societies. Rape and gender-based violence is influenced by masculinity and femininity concepts, the former identified with power. Dominance, rather than sexual gratification, can lead to sexual violence, which could also be a “male backlash” against gender equality. Biological theories link sexual violence to genetic factors; a psychodynamic perspective suggests an unconscious social reproduction of masculine culture and delves into possible explanations for violent behavior. Acculturation strategies and acculturative stress are explored, with a focus on Berry’s strategies and on Camilleri’s model of identity in intercultural situations. The impact of cultural factors on responsibility is discussed, highlighting variability in criminal laws and attitudes towards cultural offenses in different countries. The analysis of two criminal cases accused of rape, revealed common and diverging elements. Both individuals come from favorable socio-economic backgrounds, and lacked of prior or present psychiatric diagnoses. Integration difficulties, psychosexual attitudes, and the improper application of cultural codes played a crucial role. In conclusion, anthropological and ethnopsychiatric knowledge should be integrate into forensic assessments. Early detection of non-acculturation elements is need to prevent criminal behaviors, and a diagnostic instrument as a validated rating scale should be implemented.

## Introduction

According to the World Health Organization (WHO), sexual violence includes “all sexual acts or attempts to obtain a sexual act, unwarranted sexual commentary, or unwarranted sexual proposals, directed against a person’s sexuality coercively, regardless of the relationship with the victim, in any context, including, but not limited to, at home and at work” (1).

Beyond sexual gratification itself, sexual violence is often the result of a power equation between the aggressor and the victim, strongly influenced by cultural and individual values. Kalra et al. observed that cultures described as “feminist” propose equal power between men and women, and sexual violence is more likely to occur in cultures that favor beliefs in the social and cultural superiority of men (2).

It is likely that the concept of “coercion” in the WHO definition is not understood in the same way by different cultures. In fact, culture influences the definitions and descriptions of what is “normal” or “psychopathological”. Some cultures condemn various forms of violence but may tolerate others; this “cultural legitimization” gives rise to a continuum with transgressive coercion. For example, in South Africa, only the rape of white women is prosecuted, while rape of black women is not. Similarly, sexual violence is considered legitimate by young men in South Africa who also believe that mental health is negatively affected by a lack of sex (3). Additionally, in some rural parts of India, marriage involves intimate relations with children or girls. Despite sexual coercion being considered illegal, the entire issue is sanctioned by personal laws defined by those participating in such marriages (4).

The perceived consequences of sexual assault vary across cultures: in “socio-centric” societies, where individual identity is dependent and inseparable from the family, shame is a prevailing emotion, and victims of sexual assault tend not to report their experiences. In contrast, in “ego-centric” societies, where the relationship with the “self” is central, and independence is more important than interdependence, the predominant emotion is guilt, experienced intimately and privately (5). Sociologist Scheff notes the division made in anthropology between shame cultures and guilt cultures, with traditional societies relying on shame for social control and modern societies on guilt (6). Anthropologist Kroeber has also described the two emotions as follows: “shame is partly externalized; it is a feeling in relation to others; the sense of sin, however, is internal. One can feel sinful in solitude, about an act involving no harm to others. Shame as a deterrent and social force is probably in effect in almost all cultures.” (7). Therefore, it is possible that men from a sexually conservative culture might interpret non-sexual behavior or platonic interest from a woman of a sexually non-conservative culture as having sexual content, leading to sexual assault. While these theories play a major role in explaining the dynamics underlying sexual violence, they are not likely to fully account for this phenomenon.

Moreover, even if violence perpetrated by men against women represents the most common circumstance, it should be considered that gay-to-gay violence is becoming more and more common; however, few studies exist dealing with this issue (8–10).

## Methods and objectives

The present work aimed to a conceptual analysis of bias related to culture and migration in sexual assaults, including a literature review of relevant concepts and two case studies.

A search of sources was conducted in PubMed, PsychInfo and Scopus from inception to January 2024. Moreover, a snowball search and a retrieval of relevant books on the topic (based on the education of the authors) were realized.

The search included combinations of the following terms: “sexual violence”; “rape”; “cultural bias”; “penal responsibility”; “cultural adaptation”; “immigration”; “sexual coercition”; “legal framework”; “sexual coercion”; “origins of violence”; “recidivism of sexual violence”; “sex offenders”; “acculturation”; “incest”; “gender-based violence”.

The PEO framework (11) was adopted to design the present study. The population of interest includes individuals involved in sexual violence cases, focusing on the perpetrators and their cultural backgrounds. We considered diverse cultural origins and levels of acculturation within Switzerland and other relevant socio-geographic contexts. The exposure of interest is the impact of cultural origin and acculturation processes on sexual violence, especially within forensic psychiatric evaluations. This includes cultural biases influencing perceptions of coercion, responsibility, and appropriate legal and therapeutic responses. The primary outcomes of the study are the identification and analysis of cultural factors influencing: (a) the incidence and dynamics of sexual violence; (b) forensic assessment of perpetrators; (c) legal and therapeutic interventions; and (d) the psychological consequences of sexual assault within diverse cultural contexts.

From the ethical consideration standpoint, the risk associated with the included studies is low, as they predominantly consist of observational research, of anonymized case reports or purely reflect the author’s perspective. In contrast, the benefits are substantial, offering valuable insights into a pressing topic.

Broad inclusion criteria were applied for articles reviewed and discussed, which considered all sources directly addressing the impact of culture and migration on sexual violence, and possibly related to forensic psychiatry. Conceptual analyses, perspective papers, case reports, surveys, cohort, case control and qualitative studies and reviews were comprised, with no time limits, from inception to November 2024. Articles published in English, French, Spanish, German or Italian were considered.

For assessing the quality of the observational studies cited the Strengthening the Reporting of Observational Studies in Epidemiology (STROBE) (12) checklist was used, revealing a good quality, as indicated by the fully compliant or partly compliant status for most of the items. **Supplementary Table S1** reports the detailed scoring.

In our work, we initially deal, in the paragraph “*roots of violence*”, with the origin of violent, sexual and non-sexual behaviors, embracing biological, psychosocial and psychodynamic perspectives.

In the paragraph “*migration and acculturation*” we deal with migration, integration and acculturative processes, that are often related to violent behaviors linked to a mismatch between the culture of origin and the one of the host society. A focus is also present on individual strategies aimed to preserve personal identity in the migration process, and on cultural factors influencing the legal orientations on the penal responsibility, across different countries.

Last, two emblematic case reports are reported, and discussed in the light of the abovementioned theoretical background.

## Roots of violence

### Rape and gender-based violence

In any sociocultural context, the meaning of being a man/woman and of “masculinity/femininity” vary. Often, masculine identity is associated with a sense of power, and paternalistic culture encourages the idea that men protect women from “evil,” as women were largely incapable of protecting themselves.

Besides violence, the perpetration of sexual violence involves elements of control, power, dominance, and humiliation. To gain power over their victims, perpetrators of sexual violence resort to practices such as abduction, isolation, and manipulation. In this context, offenders may not necessarily find the sexual act gratifying; rather, it is the sense of power that matters (13, 14).

It has also been proposed that gender equality increases sexual violence in the form of a “male backlash.” In her seminal work on the intercultural aspects of heterosexual rape, Sanday found that rape is crucial in sociocultural configurations centered around male domination and the “ideology of toughness”, that can be shared by both men and women (15).

Briere et al. examined the self-reported proneness to rape or sexual coercion in a cohort of 352 male psychology students (N=352). The likelihood of rape and coercion were predicted by pro-rape attitudes and by a combination of pro-rape attitudes and sexuality variables (e.g. sexual experiences, the importance of sex, a current relationship with a women, use of pornography) but not by sexuality variables alone (16).

Jaffee and Strauss indicated that there is no relationship between sexual attitudes and sexual violence but instead a significant association between urbanization, poverty, divorce and sexual violence (17).

In our opinion, rape can also be seen as the psychological extension of a sex-stereotyped culture, where sociocultural attitudes transmitted to women, rapes, and rapists can predict sexual violence, and these stereotypes are often internalized in male-dominated sociocultural environments.

Sexual violence can result from a widespread misogynistic attitude in a culture, and it has been observed that in patriarchal cultures, the victim’s resistance is often experienced by the aggressor as an insult to his “masculinity,” leading to an increase in violence aimed at controlling the victim (18).

Other factors should be considered, specifically predisposing to gay-to-gay sexual violence. Concerning men-vs-men sexual violence, Javaid proposed that heteronormativity (i.e. the normalization of heterosexuality as the sole acceptable sexual orientation) creates a social landscape where male rape is misunderstood, minimized, or even dismissed (19). Ayhan Balik et al. concluded that a higher level of outness (i.e. openly disclosing one’s sexual orientation), perceived discrimination, and internalized homophobia favor the perpetration of woman-to-woman sexual violence (9). Last, a report on the LGBT community indicated that barriers to seeking help such as the fear for outness and the lack of trust in law enforcement were predisposing factors for being victim of an intimate partner sexual abuse (10).

### Biology versus culture

Sexuality is influenced by genetic factors, but its expression also reflects the cultural background (20).

The biological theory of sexual violence proposes that it is the byproduct of an early, evolutionary selected, adaptation process for fitness (i.e. a measure of the success of an individual with a given genotype in bear an offspring). In this perspective, men’s sexual urgency would be an adaptive process leading to a “reproductive strategy” including sexual violence, to impregnate as many women as possible. This theory implies that every man has an innate propensity for sexual aggression and violence, rooted in the genetic background.

Other theories explain sexual violence through a sociocultural model and deny the biological foundations of sexual impulses: power dynamics between genders, moral values, and attitudes toward violence are more influential on sexual behavior. In this regard, Sanday discusses two types of cultures: “rape-free” (exempt from rape) and “rape-prone” (susceptible to rape), which are shaped by sociocultural values. The former would be characterized by gender equality, while the latter would exclude women from positions of power, limit their freedom and consider them as objects (15).

Otterbein examined seventeen different societies, noting that cultures with a rigidly defined gender role system exhibit a higher level of sexual violence, which would be the consequence of male power (21).

Thornhill et al. combined the genetic and the socio-cultural hypotheses, arguing that socially learned behaviors have a biological base, and that an overlap of biological and cultural causes occurs in sexual violence (22).

### Psychodynamic perspective

Bourdieu refers to “symbolic power” as the unconscious social reproduction of a masculine culture, accepted by both the dominated female and the dominant male; this perspective emphasizes power dynamics and assume men as the natural holders of power within relationships. Consequently, their violent behavior would legitimately aim at possessing the partner or ex-partner. On an individual level, aggressors are often depicted with a certain fragility, pathological narcissism, communication and reasoning difficulties, as well as emotional and interpersonal self-regulation deficits (23).

For individuals in such circumstances, it is crucial to self-regulate internal threats of annihilation, closely linked to murderous/suicidal fantasies. In an analytical biopsychosocial approach, much of violent behavior is rooted in early affective experiences (13), with violence deriving from the failure of the psychic integration process. According to the psychoanalytic model, this failure leads men to attribute a split part of themselves to their partners through projective identification. Violent adults use this mechanism as a psychological defense, due to an inability to handle and mentalize their emotions. The aggressed person become a repository for these parts of the violent person, to the point that every non-response to aggressors’ needs threatens their sense of self and cause self-disintegration. This mechanism creates a context that is hostile to women but keep them tied to their partners (14).

An approach proposed by Johnson theorized the existence of two forms of violence in couples: “patriarchal terrorism,” descending from cultural stereotypes, and “common couple violence,” which arises from family dynamics. In this model, “patriarchal terrorism” is a product of the social tradition where men feel entitled to control women through violence, subordination, and isolation. It is worth noting that conflicts that arise between partners in a couple are often framed within a patriarchal context, even when they do not lead to violence (24).

**Figure 1:**
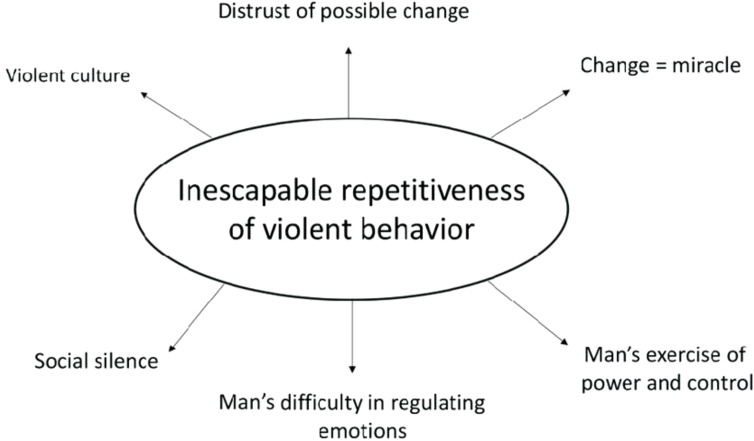
Procentese’s theory on the inevitable repetition of violent behaviors.

Hearn defines the causes of violence by considering three levels: individual, interpersonal level (such as socialization and learning within the family and other relationships) and broader sociocultural aspects related to power (25). In this context, the study by Procentese et al. (14) illustrates (**Figure 1**) the basic category and the semiotic network of the inevitable repetition of violent behaviors, emphasizing the role of social silence, which contributes to tolerance for violence, and of a culture of violence already inherent in the family functioning of individuals who act through violent modalities.

## Migration and acculturation

### The subject of the migration process: definition

The International Organization for Migration (IOM) defines an international immigrant as a non-national person who enters a country with the intention of settling. Other authors use more general definitions, for example, a person who has established a new permanent (semi-) residence in a “place” other than where they usually lived. Often, the terms immigrant and foreigner or born abroad are used interchangeably. However, different ways of classifying immigrant status can lead to disparate results in studies conducted on these populations.

Two main definitions are found in the literature:

1. “Born abroad”: a person born in a country other than their current country of residence. This defines an unchanging status but excludes second and third generations of immigrants since it is independent of the migratory status of their parents/grandparents.

2. “Foreigner or non-national”: a person belonging to another state or owing allegiance to it. This status can vary over time and depending on the legal requirements of each state, which often depend on historical ties between countries (26).

Kyrkmayer et al. classified stress factors associated with migration and resettlement, influencing mental health (see **Table 1**). The “healthy immigrant effect” reflects the filtering process individuals must undergo to obtain immigrant status. However, factors related to the post-migration phase can moderate the effects of the pre-migration protective factors, leading to a negative outcome. For example, Canadian immigrants initially have a slightly lower rate of mental disorders than the general Canadian population, but this rate increases over time and becomes similar to that of the general population (27).

**Table 1:** Common mental health problems in immigrants and refugees: general approach in primary care, Canada Medical Association (27).

Premigration	Migration	Postmigration
Adult			
Economic, educational and occupational status in the country of origin	Trajectory (route, duration)	Uncertainty about immigration or refugee status
Disruption of social support, roles and network	Exposure to harsh living conditions (e.g. refugee camps)	Unemployment or underemployment
Trauma (type, severity, perceived level of threat, number of episodes)	Exposure to violence	Loss of social status
Political involvement	Disruption of family and community networks	Loss of family and community social support
	Uncertainty about outcome of migration	Concern about family members left behind and possibility for reunification
		Difficulties in language learning, acculturation and adaptation (e.g. change in sex roles)
Child		
Age and developmental stage at migration	Separation from the caregiver	Stresses related to family’s adaptation
Disruption of education	Exposure to violence	Difficulties with education in new language
Separation from extended family and peer network	Exposure to harsh living conditions (e.g. refugee camps)	Acculturation
	Poor nutrition	Discrimination and social exclusion (at school with peers)
	Uncertainty about the future	

The assessment of the risk of mental disorders includes the “pre-migration” factor, exposure to stressful factors and the duration and uncertainty of the migration period. Resettlement (post-migration) ultimately influences adaptation and health conditions in the medium and long term.

A complex interplay exists between physical, sexual and psychological violence, migration and acculturation. On one side, the experience of violence is common during the migration process, leaving profound psychological wounds and impacting acculturation. On the other side, a poor acculturation process may favor violence, especially in the case of behaviors that are not considered violent or that are accepted in the country of origin (28, 29).

### Acculturation and identity strategies

In cultural anthropology, acculturation is defined as “the set of cultural changes resulting from continuous and direct contact between two independent cultural groups” (30).

Berry introduced the notion of psychological acculturation, indicating the changes at the individual level that accompany contact between groups. In principle, changes between the groups involved are mutual, but it often happens that one group, typically the holder of the host culture, exerts a stronger influence than the immigrant group (31).

Acculturation in fact involves individuals adjusting to the cultural dynamics of the host culture. This process can develop through various strategies, reflecting how individuals manage their dual cultural identities. Berry identified four main acculturation strategies:

1. Assimilation: Individuals adopt the values and practices of the host culture while relinquishing their original cultural identity;

2. Integration: Individuals maintain their original cultural identity while also adopting aspects of the host culture, resulting in a bicultural identity (32);

3. Separation: Individuals prioritize their original cultural identity and avoid adopting the values of the host culture;

4. Segregation: the host group prevents any relationship with the immigrant person, leading to an outcome similar to separation;

5. Marginalization: the non-dominant group has lost its identity and is not allowed to participate in the functioning of institutions and in the life of the dominant group (due to discriminatory practices). Individuals of the non-dominant neither maintain their original cultural identity nor seek to form relationships with the host culture, leading to cultural alienation.

Berry gives importance to the pressures exerted by the dominant group, and in particular he states that integration implies that the host society allows the minority to maintain its cultural identity in the private sphere, guarantees economic and political rights but impose the respect for its fundamental values and laws (31, 33).

The option of “individualism” was described, referring to migrants who reject both the support measures of their ethnic community and those of the host society, firmly believing in a meritocratic system, having a high self-esteem and being convinced that they will autonomously achieve their goals; the position of individualism is more typical of migrants with a high level of education. Also, members of the host society can hold an individualistic position, considering individual characteristics more important than the group membership, and giving no importance to the cultural identity of the host and hosted social groups (34). In egocentric societies, as the occidental ones, individualism is likely to be a more common position.

### Acculturative stress

Acculturative stress is the result of the acculturation process itself and “manifests as mental health problems (confusion, depression, anxiety, etc.), marginality (…), alienation, and identity difficulties”; Acculturative stress can arise from challenges such as language barriers, discrimination, cultural differences, and a sense of belonging (33).

Identity strategies play a crucial role in this dynamic, and are deeply influential on the sense of self in the context of acculturation. The choice of acculturation strategy and the experience of acculturative stress can vary among individuals and are influenced by individual’s personality, social support, and the broader socio-cultural context. Developing a strong and positive bicultural identity is often associated with better psychological well-being.

Acculturative stress would be “probable and not inevitable,” meaning that its risk is moderated by adjustment and resilience factors. Culturally plural societies are characterized by networks of social and cultural groups that can provide support to those undergoing the acculturation experience and by a greater acceptance of cultural diversity (“multicultural ideology”). This is linked to the particular configuration of ethnic and racial attitudes: some acculturation groups may be more easily accepted and placed higher on a prestige scale, while others occupy lower ranks due to prejudices. As for other acculturation processes, separation implies resistance to intergroup relations, maintains conflict and involves a high degree of frustration, while marginalization represents a state of continuous crisis. Individuals in this category experience psychological and social problems due to acculturative stress, identity confusion, alienation and social deviance. Overall, all results converge to indicate that integration is the most favorable solution regarding the mental health of migrants, followed by assimilation, with marginalization being the least favorable and separation in an intermediate position. The integration strategy incorporates several protective factors: freely consensual mutual accommodation (mutually positive attitudes, absence of prejudice and discrimination), participation in two cultural communities and having a flexible personality (30, 31, 33).

### Camilleri’s model of identity strategies

The notion of identity strategy originates from the postulate that every individual seeks positive self-esteem (positive identity) and that belonging to social groups depends on it. When identity is devalued or questioned, the individual resorts to strategies to restore it. Camilleri takes up this idea as the basis for his theory of identity strategies in intercultural situations, such as migration. According to the author, the migrant experiences the contact between their culture of origin and that of the host society as a conflict, as the possibility of a cultural fragmentation affecting his identity. Camilleri states that the coherence between the ontological function of identity (i.e. related to acculturation) and the pragmatic function of identity (related to the need to adapt to the environment) is shaken.

Another upheaval the migrant must face is a damage to self-image resulting from their dominated status in the host society. According to Camilleri, migrants resort to two types of strategies to cope with this situation:

- on the one hand, there are “dependent identities”, occurring when migrants internalize a negative image of themselves, imposed by the host society, and conform their reality and values according to this “negative identity”;
- Other migrants try to deflect depreciative judgments from the host society by fully immersing themselves in the socio-cultural whole, resorting to assimilation and shifting the negative image to other members of the community of origin (“displaced negative identity”). The individual can also distinguish themselves by becoming aware of their uniqueness without internalizing devaluation(“identity by distinction”). The “reactive identities” would express the individual’s will to emancipate from the prescribed negative image by claiming their origin (“defense identity”), over-affirming stigmatized characteristics to escape depreciating feelings (“polemical identity”), or claiming membership in the original group while rejecting its values in their actions (“principle identity”) (35–38).

### Impact of cultural factors on responsibility

Bernardi defines the concept of cultural offense, as an offense strongly influenced by the specific “cultural factor” of the author, leading to behaviors prohibited by law. Examples are individuals from Central African countries who, according to their own traditions, engage in sexual relationships with their minor compatriots; native South East Asians who assign adolescents to work prohibited due to age in the country of residence; individuals who support a conception of family life where the head of the family can exert an absolute power on the other members, leading to behaviors constituting a maltreatment according the law of the host state; individuals subjecting men and women to ritual mutilation or deformation (circumcision, ornamental scars, elongation of the neck or lips, foot binding, infibulation, etc.).

It is noteworthy that national criminal laws vary and the attitude of different countries toward cultural offenses is more or less lenient depending on whether the countries in question are characterized by a *“multicultural”* social and political model (as in Anglo-Saxon countries) or by an “*integrationist”* model (as traditionally seen in France). Specifically for the United States, the United Kingdom, Australia, Canada and South Africa, there is a significant development of what is called “cultural defenses,” based on which the commission of certain criminal prohibitions, when “culturally motivated,” may result in a reduced criminal responsibility. On the contrary, Switzerland and France have chosen not to give any importance, at the criminal level, to the possible affiliation of the author to a community with deeply different cultural roots (39).

Models mixing aspects of the multicultural and the integrationalist ones are emerging in Western countries, where the role of the cultural factor in the criminal domain is evaluated differently depending on the case (i.e., according to the current political options, the type of offense committed and the demands from the social body against it). Notable cultural differences with significant repercussions in criminal matters can be observed between subjects coming from culturally and geographically close societies. An example is the significant difference in tolerating drug use between France and the Netherlands. The same applies to driving at high speeds by a German on a Swiss or Danish highway due to the absence of speed limits on German highways and, conversely, the presence of very strict limits in the neighboring Nordic countries. On this subject, Bernardi concludes that the “car culture” of the German driver prevents him from fully understanding the illicit nature of his own behavior. Coming to culturally based criminal prohibitions, examples are: assaults and family abuses committed by persons from countries where such abuses are part of the “ius corrigendo” of the head of the family; polygamy practiced by an immigrant born in a country where having multiple wives is licit; begging while accompanied by a minor, thus preventing him from attending compulsory school. According to Bernardi, while the objective severity equal to that of the same illicit acts when not supported by the traditions of the group of origin, the subjective severity is different depending on whether the subject has or has not committed the offense based on a “predisposing cultural factor.” (40)

## Case reports

Subjects undergoing forensic evaluation may not have a psychiatric pathology as defined in an official diagnostic system such as the ICD or DSM; at the same time, they may exhibit signs that a process of acculturation or integration has not occurred or is not complete. These “cultural signs” can be prodromal or precursors to mental disorders, which could relate to offense committed.

Although clinical observations related to migration, integration or social maladjustment are frequently recorded in the context of a forensic report, there is still no standardized system for their objective evaluation. This makes it challenging to use them for forensic purposes, especially concerning subject’s responsibility, dangerousness and criminal recidivism.

Here, we present some data concerning two forensic cases, conducted on young men who were accused of the rape of a woman. Each of the two forensic works involved three one-hour interviews and the administration of the risk assessment scale Sexual Violence Risk-20 (SVR20) (41).

The content of the two case reports was validated based on the concept of trustworthiness (42). Credibility (i.e. the confidence in the truth of the findings) was given by authors’ experience on the topic, within the tradition of their institution, and by applying the principles of prolonged engagement (i.e. spending adequate time in the field, building rapport with the cases to understand their perspective) and reflexivity (i.e. acknowledging personal biases and preconceptions throughout the evaluation process); moreover, clinical cases were handled by a team of forensic psychiatrist, and regular debriefings and supervisions on the cases were performed. Dependability (i.e. the stability of data over time and under different conditions) and transferability (i.e. the extent to which findings from a study can be applied or transferred to other contexts, settings, or populations) were guaranteed by the structured clinical assessment, carried out according to a rigorous interviews’ schedule and using standardized key questions on the personal and psychiatric history and on the sexual violence.

### Personal history

- Mr. N.: a man who, at the time of the alleged acts (in 2021), was 23 years-old. His parents are of Russian origin and the individual was born in Vienna, where he reportedly lived for the first two years of his life before going to Russia for about two years, before moving for a few months in South Africa and, for about 7 years, in the Netherlands. He immigrated to Geneva in 2008 with his entire family. Mr. N is the eldest of 4 siblings. The individual’s parents are said to be diplomats. The experts identified a very strict family upbringing, characterized by acts of physical violence endured by the individual for educational purposes. Mr. N mentioned that until the age of 10 or 11, he used to receive corrections from his parents with a belt, specifying that, at that time, it was something more common than it is now; however, he also noted that his parents would have never done the same to his two younger siblings. Mr. N was accused of raping his 17 years old sister. After his sister filed a criminal complaint, Mr. N. reported that he became a very practicing Christian again, as suggested by his father, to atone for his guilt.
- Mr. D.: a man of 25 years old at the time of the alleged acts (in 2022). He is of Senegalese origin and immigrated to Geneva in 2019, where he joined his mother and sister, who had already been living there for several years, while his father and second sister were remaining in Dakar. Mr. D. was the eldest of three-siblings. Mr. D was born from parents of Senegalese nationality, with the mother working as a diplomat in Switzerland and the father living in a large family home in Senegal, where he was involved in livestock and agriculture. Mr. D. claims to have moved to Switzerland with his younger sister to pursue his dreams and goals, namely obtaining internationally recognized diplomas. He grew up with the basics of the Muslim religion, and he mentioned that it played a relevant role in his education in Senegal. He was following its rules rigorously, even when in doubts about their validity. He claimed to have acquired a more “objective” view on the subject and a “discernment capacity.”, being now more grown up. In fact, he explained to the experts that he found “contradictions” in religion, pointing to the example of terrorists who, in the name of religion, advocate violence. Furthermore, he stated that he could not, in Senegal, adhere to abstinence from substances such as tobacco and cannabis and practice religion as his mother wanted (for example, by performing the five prayers and observing Ramadan). His arrival in Switzerland would have finally convinced him to not to practice religion, although this generated a conflict with his mother (“she wanted me to be a practitioner but I have an objective vision.”). Mr. D was accused of raping a woman known through a dating app. Mr. D. describes his society of origin as very strict and adhering to rules with a strong Muslim influence, and he reported a total break from since arriving in Geneva.

### Socio-economic context

Both individuals came from a favorable socio-economic background and were not known for any psychiatric diagnoses prior to the alleged acts, or for hospitalizations in psychiatric settings.

- Mr. N.: regarding alcohol consumption, Mr. N. denies ever having a problematic consumption, reporting an occasional use of wine or beer during social gatherings. However, he recalled an episode in which he had his driver’s license suspended due to a road accident that occurred while he was intoxicated. Concerning the accusations of his ex-girlfriend, stating that he used to force her to drink alcohol, he denied any form of obligation, and he held that he use to fill glasses for women for a form of politeness. Regarding other substances, Mr. N. recognized having used cannabis during university, until the summer of 2019, driven by the feeling to be more creative when smoking. He also mentioned using cocaine to enhance his professional performance when he worked to pay for a semester of university (that he actually failed). Finally, he claimed to have stopped all consumption overnight.
- Mr. D.: Mr. D. said he drank alcohol for the first time in Geneva because he had no access to it in Senegal, being a Muslim country where alcohol is prohibited. He stated that he would primarily drink to “sync with others,” sometimes combining it with cannabis. He stated that the real reason for his incarceration was his mother’s accusation of using cannabis, which would have been “unacceptable to a Muslim woman”. He reported the feeling to be not understood by her mother, and he banalized the fact that she reported him for domestic violence (mostly verbal), while he was under the effect of cannabis; he stated that it was just a trivial dispute occurring while he was slicing meat during Ramadam.

### Attitudes toward accusations

- Mr. N.: reported having no memory of sexual acts committed with his sister. He only recalled waking up next to her, and declared being against any form of incest. He expressed the need to believe that nothing happened, being difficult to him to accept to be responsible for the nature of the alleged acts. He emphasizes being drunk and under the effect of ecstasy
- on the night of the incident. He questioned his ability to have a sexual relationship under the influence of drugs. Mr. N. mentioned that the forced isolation from the rest of the family following his accusation was almost unbearable.
- Mr. D.: being incarcerated, he emphasized the consequences on his university ambitions and the “dream” of building a career in the immigration country Switzerland, while showing little interest in the alleged victim. He held a contemptuous view toward her, criticizing her “punk” and “gothic” dressing style, her disorderly living conditions and her poor cooking skills. He reported being insulted by the victim, being unable to acknowledge the disproportion between the supposed insults and his act When asked about his understanding of the concept of consent, he confidently asserts that “certain things are not asked in action” and shows difficulties in putting himself in the other person’s shoes. For example, he can explain that, at his young age, it is important to sexually blossom through various conquests but would not accept if his partner did the same.

### Integration difficulties

Experts detected integration difficulties that occurred early upon the individuals’ arrival in the Swiss community.

- Mr. N.: confronted with his restrained emotions’ expression, Mr. N. replies that the cause was his difficulty in expressing himself in French. He specified that he feels emotions internally, but he is not accustomed to showing all of them and believed that their expression must be restrained in public. He stated that he would more easily show emotions like joy or anger, while sadness was not something to share, in line with what he learnt from his parents and family. Mr. N. highlights that his integration in Switzerland was difficult due to social isolation, and that he was sometimes talking to himself. He reported having experienced mockery from girls of the same age due to his small stature and acne in the period following his arrival in Switzerland. However, Mr. N. does not recognize that this experience created a sense of vengeance in him.
- Mr. D.: Mr. D. explains that he regularly participated in household duties by cleaning or tidying up the house but did not cook because the religious tradition of his country would dictate that “men do not cook but do household chores and shopping.” The main goal of his transfer to Switzerland was to achieve academic results that will lead him to a successful professional career; however, he started the university during the Covid-19 pandemic, forcing him to attend online classes. Being isolated and non-integrated with his peers, he started using dating applications.

### Psychosexual attitudes

- Mr. N: he considers sexual relations to be something very intimate and emphasizes in the importance of mutual trust with the partner, refusing “physical-only encounters.” He acknowledges having previously used the services of a prostitute at the age of 19.
- Mr. D: in Switzerland, he mentions a greater freedom to enjoy his sex life, considering the country to be less constrained by religion. He sees any physical relationship as consensual, motivated by mutual “feeling” and based on respect for the other person. For the relationship to work, “she must respect me.” The evaluated person specifies his view of women, stating that “women should complement men psychologically.” He acknowledges having used the services of prostitutes five times since his arrival in Switzerland. He assures that sexuality is a “taboo” subject in a Muslim country like Senegal: “it is practiced, but it is not talked about, it is not learned.” He explains that he never received sexual education courses during his schooling. In Switzerland, Mr. D. mentions greater freedom to enjoy his sex life, because of a different culture compared to his land of origin. He says he sees any physical relationship as a consensual connection motivated by mutual “feeling” and based on respect for the other. Thus, for the relationship to work, “she must respect me.” The assessed person specifies his view of women by stating that “women must complement men psychologically.” He reported to have used the services of prostitutes five times since arriving in Switzerland

### Forensic evaluation

In the evaluation of historical and clinical data, no psychiatric diagnosis was established for the two individuals. Criminal responsibility, in the event of guilt, was assessed as full in both cases. The risk of recurrence was assessed as low and low-medium, for Mr. N. and Mr. D., respectively. In the assessment of dangerousness, the score Mr. N. obtained on the SVR-20 scale, under the assumption of guilt of the assessed person, was 5/40. It emerged from the evaluation of the risk of recurrence of sexual violence that the assessed person does not suffer from any sexual or psychiatric disorder, addiction, has no criminal history and is adequately integrated socio-professionally. Compared to other authors of sexual violence, the assessed person has few risk factors for recurrence. For Mr. D., the score obtained on the SVR-20 scale was 7/40 and, assuming the facts are proven, this result was weighed against the clinical evaluation conducted by the experts. No therapeutic measures were recommended for the assessed individuals, suffering from no mental disorders.

## Discussion

In the two cases considered, common and diverging elements are found: Mr. N. manifests his affects in a very intimate way, with a tendency to control close persons, especially women, through a subtly dominating attitude stemming from his parental upbringing. He describes himself as the pillar of the family, with a father’s vicar role, in line with his Christian and paternalistic culture. Following the alleged offense and on his father’s suggestion, he intensively addresses it in order to expiate his guilt despite the low empathy towards the victim, his sister, described as the fragile element of the family.

In the case of Mr. D., Islam, to which the assessed person rebels, plays a central role. Mr. D. considered obsolete many precepts of his origin culture, such as the prohibition of eating certain meats, consuming drugs, or freely addressing the subject of sexuality. However, he maintained a “macho” spirit towards women and their roles in the family and the society, and justified his act by devalorizing the victim, because a “inappropriately dressed, bad cook”, features making her a weak and unreliable subject, not deserving to “complete him” psychologically.

In the case of Mr. N., the “cultural distance” from the place of insertion (Switzerland) is apparently less pronounced than in Mr. D. Mr. N.’s social functioning seems high and he is strongly supported by his family of origin, even after the offense. However, during the “post-offense” phase, he does not seek individual answers to his actions, but finds solutions that are once again deeply embedded in his beliefs of belonging (for example, to pray as a method of expiation). He shows no form of self-questioning about the facts, relying on the fact that his sister is now a psychiatric patient, subjecting her to stigma. In contrast, Mr. D. complains that his mentality of origin is “extremist,” even rich in moral prohibitions. Although he has partly internalized the macho elements of his culture of origin, he tries to break away from it. This process is facilitated by his distance from the family, which increased after the offense. In both cases, we note the presence of difficulty in adapting to the Swiss system, to which both assessed individuals try to conform by improperly applying the cultural codes of the new country.

In Mr. N., we observe a dimension of power and control over women that mirrors the paternal male figure, in line with the idea that the eldest son is required to assume a patriarchal role. Mr. N has experienced humiliation from being mocked by girls (due to his physical appearance) and being unable to establish an equal relationship with females, resulting in a threat to his masculinity. Mr. D. believes that women have certain given social and emotional duties to be a good partner; if a woman does not adhere to these standards, she only is suitable for a mere sexual interaction. Despite the maternal emancipation, his cultural background is markedly patriarchal: a man must be cared for and pampered (through good cooking, home management, etc.). The masculine humiliation here lies in not receiving from women what is due to him as a man (13–15).

In this work, no biological aspects (43) were identified that play a primary role in sexual violence. Instead, it is noted that both cultural backgrounds of the individuals studied are characterized by ‘rape-prone’ values (15): in the case of Mr. N., this is more covert (through a vicarious paternalistic-dominant role), while in the case of Mr. D., it is expressed in a more explicit manner.

According to a psychodynamic perspective, in the case of Mr. N., sexual violence represents a way of asserting power, aimed to stabilize fragile intrapsychic dynamics; it is therefore a self-regulation mechanism related to narcissistic impulses (23). The fact that the victim is the perpetrator’s sister confirms the need to reestablish patriarchal hierarchies within the family (24).

Mr. D. expects that women, even in the new Swiss social system, will share his mentality and accept their fate as ‘the dominated.’ According to him, men are the natural holders of power, as observed in both his romantic interactions and in those with his mother and sister (20, 21).

Mr. N.’s acculturation strategy corresponds to an assimilation as he appears to have adopted the values and practices of the host culture, abandoning his original cultural identity, in a process linked to performance rather than to feelings of shame (31–34). However, A regression to a ‘separation’ model occurred in the period following the offense (for example, seeking refuge in prayer, on paternal advice, as the only strategy for atoning for guilt).

In the case of Mr. D., it is observed that the main acculturation strategy lies between separation and marginalization (31, 32): his cultural identity from his origin is experienced as a priority and is characterized by an avoidance of the values of the new culture, from which he nevertheless wishes to derive benefits (such as easier access to sex and psychoactive substances). Mr. D.’s acculturative stress manifests as frustration, which leads to inadequate reactions towards his family of origin, anxiety, and progressive social isolation.

According to Camilleri’s model (35–37), we can observe that Mr. N.’s strategic identity tendency is to deflect disparaging judgments from the host society and fully immerse himself in the socio-cultural fabric, resorting to assimilation. There is a claim of belonging to his group of origin but at the same time a rejection of its values in his actions (‘principled identity’). On the other hand, Mr. D. resorts to a ‘polemic identity,’ hyper-expressing the characteristics stigmatized by the host society in order to escape devaluing feelings and enacting a rejection of the negative identity.

The analysis of the two clinical cases, supported by the theoretical background we discussed, allow to draw several conclusions on the impact of cultural factors on the risk to commit a sexual violence, and on the forensic psychiatry practice.

The interplay of power, control, and gender violence reflects deeply entrenched patriarchal values that act as significant catalysts for sexual violence. The concepts of entitlement and humiliation illustrate that individuals may resort to violence as a misguided assertion of masculinity within a framework that mandates women to fulfill specific social roles. From a psychodynamic perspective, these violent behaviors often serve as maladaptive mechanisms for individuals to stabilize fragile self-structures, manifesting as projections of intrapsychic conflicts.

Moreover, the processes of migration and acculturation can develop maladaptively, indirectly fostering environments that may legitimize or escalate sexual violence. This acculturative stress complicates identity formation and can exacerbate feelings of frustration and disconnection from social norms.

While these factors are not strictly psychiatric, they are vital in forensic evaluations, emphasizing the need for interventions that address the underlying cultural and psychological dynamics contributing to gender violence. Recognizing the significance of these elements can provide a more comprehensive understanding of the motivations behind such behaviors, highlighting the importance of broader cultural considerations in forensic practice. In particular, the early detection of non-acculturation or low cultural integration elements could prevent behavioral drifts.

In principle, in the absence of an established diagnosis, experts do not recommend therapeutic measures. However, if we consider that low cultural integration is a risk factor for criminal recidivism and for psychiatric disorder, it would be indicated to propose a psychotherapeutic treatment and a clinical follow-up. The use of psychotherapy as a facilitator of the integration process is also thorny because, while it can facilitate the development of awareness of intrapsychic and environmental phenomena during the individual’s life, it will not consider the group identity of the community where the subject lives, typically characterized by a socio-centric functioning. The limitations of these proposals are certainly related to the claim to objectify, through a standard instrument and limited variables, a complex theme that has deep roots in personal and societal history and constantly exposed to dynamic events. There is a concrete risk of simplifying the reading of the integration process as well as of trivializing the role of subjective identity. It is also necessary to question whether the presence of anthropological and ethnopsychiatric knowledge in the expert is not essential for the expert’s purposes. Specific knowledge could in fact facilitate the understanding of the acculturation process and, in the end, the offending dynamic and its stakes.

This paper presents some strengths and limitations. Strengths are represented by the interdisciplinary approach, including insights from various fields, including psychiatry, psychology, anthropology sociology and the law; the paper effectively contextualizes the issue of sexual violence by exploring its roots in broader social, cultural, and political structures; it combines clinical data from two criminal cases with a conceptual analysis, linking concrete examples with a theoretical reflection.

Limitations are as follows: first, the theoretical context is not exhaustive, as there is limited literature on this subject. The selected themes and perspectives on sexual violence are inherently restricted, impacting the reach of our findings; however, this can be justified by the novelty of the topic under analysis, and it makes it appropriate the editorial form of a perspective paper. Additionally, our analysis is based solely on two case reports, and a larger study would have provided more significant insights; however, this innovative approach initiates essential discourse in the field. While the cases originate from two different cultures, the cultural contexts are relatively similar, which may limit the ethnic-psychiatric perspective. Finally, the paper mainly addresses male-to-female violence, since existing literature predominantly covers male-perpetrated violence against women, and only few studies addressed the topic of gay-to-gay sexual violence (8–10). While these studies offer relevant knowledge about prevalence and risk factors, they cannot be considered exhaustive when it comes to purely psychiatric or psychological aspects. In fact, the assessment of potential perpetrators’ mental disorders is lacking, and the proposed theories on the psychological and societal underpinnings of violence need to be further corroborated; moreover, sexual violence in these papers is not related to the topic of the psychiatric expertise, missing the link with the main focus of our investigation.

Authors retrieved no articles dealing with the topic of female-to-men violence, warranting investigations on this specific topic.
