# Supplementary material for: The impact of cultural origin on the psychiatric expertise in Switzerland: a focus on sexual violence illustrated by two criminal cases
Source: Front Psychiatry. 2025 Jan 28;15:1390224. doi: 10.3389/fpsyt.2024.1390224 (PMC11811092; doi:10.3389/fpsyt.2024.1390224)
Supplement: Supplementary file 1 [file Table1.pdf]

Supplementary table S1: STROBE checklist for observational studies

		Sanday 1981	Briere 1983	Jaffe 1987	Berry 1989	Krahé 2000	Brown 2015	Gimenu-Feliu 2019	Ayhan-Balik 2019	Otterbein 2020	Procentese 2020
**STROBE ITEM**	1	1	1	2	1	2	1	1	2	2	1
	2	2	2	1	2	2	2	2	2	1	2
	3	2	2	2	2	2	2	2	2	1	2
	4	1	2	2	1	2	2	2	2	1	2
	5	1	2	1	2	1	2	2	1	1	1
	6	1	2	Na	2	1	2	1	2	1	2
	7	2	1	2	2	2	2	1	2	1	2
	8	1	2	2	1	1	2	1	1	2	1
	9	0	0	0	0	1	0	1	0	0	0
	10	1	0	0	0	0	0	0	0	0	0
	11	1	1	1	1	1	2	2	2	1	2
	12	0	0	1	1	1	1	1	2	1	1
	13	1	1	Na	2	1	1	1	1	1	1
	14	2	2	Na	1	2	1	1	2	1	2
	15	1	2	2	2	2	2	2	2	1	2
	16	2	2	2	2	2	2	2	2	2	2
	17	Na	Na	Na	Na	Na	Na	Na	Na	Na	Na
	18	2	2	2	2	2	2	2	2	1	2
	19	0	0	0	0	2	2	2	2	1	0
	20	2	1	2	2	1	2	2	2	1	2

	21	1	1	1	1	1	2	1	2	1	1
	22	0	0	0	0	0	2	2	2	1	2

Studies are rated 0, 1, 2 if non-compliant, partly compliant or fully compliant with the STROBE recommendations. Na not applicable.
